# Video head impulse testing in patients with isolated (hemi)nodular infarction

**DOI:** 10.3389/fneur.2023.1124217

**Published:** 2023-02-06

**Authors:** Seung-Han Lee, Jae-Myung Kim, Joon-Tae Kim, Alexander Andrea Tarnutzer

**Affiliations:** ^1^Department of Neurology, Chonnam National University Medical School, Chonnam National University Hospital, Gwangju, Republic of Korea; ^2^Department of Neurology, Cantonal Hospital, Baden, Switzerland; ^3^Faculty of Medicine, University of Zurich, Zurich, Switzerland

**Keywords:** cerebellum, acute stroke, vestibulo-ocular reflex, acute vestibular syndrome, episodic vestibular syndrome, vHIT

## Abstract

**Background:**

Isolated (hemi)nodular strokes as underlying cause of acute dizziness are rare, thus there are still gaps of knowledge in the clinical presentation of affected patients. Clinical and experimental evidence has suggested that lesions involving the nodulus lead to various vestibulo-ocular deficits including prolonged velocity-storage, periodic-alternating nystagmus, positional nystagmus, abolished suppression of post-rotatory nystagmus by head-tilt and impaired verticality perception. At the bedside, the angular vestibulo-ocular reflex (aVOR), as assessed by the horizontal head-impulse test (HIT), has been reported to be normal, however quantitative assessments of all six semicircular canals are lacking.

**Objective:**

The primary aim of this case series was to characterize the spectrum of clinical presentations in isolated (hemi)nodular strokes. Furthermore, based on preliminary observations, we hypothesized that the aVOR is within normal limits in isolated nodular strokes.

**Methods:**

We retrospectively included patients with isolated (hemi)nodular stroke on diffusion-weighted MR-imaging from a prospective stroke-registry. All patients received a standardized bedside neuro-otological assessment and quantitative, video-based HIT (vHIT) of all six semicircular canals. Overall ratings of vHIT (normal vs. abnormal function) were performed independently by two reviewers and disagreements were resolved.

**Results:**

Between January 2015 and December 2021 six patients with isolated nodular (*n* = 1) or heminodular (*n* = 5) ischemic stroke were included. Clinical presentation met diagnostic criteria for acute vestibular syndrome (AVS) in 5/6 patients and for episodic vestibular syndrome (EVS) in 1/6 patients. Ocular motor abnormalities observed included the presence of spontaneous horizontal nystagmus (*n* = 2), positional nystagmus (5/6), head-shaking nystagmus (3/6), skew deviation (*n* = 1), and moderate or severe truncal ataxia (5/6). Bedside HIT was normal in all patients and no gaze-evoked or periodic alternating nystagmus was observed. aVOR-gains were within normal range in all patients and overall aVOR-function as assessed by vHIT was rated as normal in all six patients.

**Conclusions:**

Using quantitative, video-based testing of the horizontal and vertical aVOR, preserved integrity of the aVOR in (hemi)nodular strokes was confirmed, extending preliminary findings at the bedside. Furthermore, widespread deficits of both ocular stability, postural control and volitional eye movements were observed in our study cohort, being consistent with findings reported in previous studies.

## 1. Introduction

The nodulus, i.e., the most caudal part of the cerebellar vermis (lobule X) is essential in the processing of vestibular information (1). Together with the flocculus, the paraflocculus and the ventral uvula it constitutes the vestibulocerebellum (2). Clinical and experimental evidences have suggested that lesions involving the nodulus lead to various vestibulo-ocular deficits. Specifically, the nodulus and the uvula control the time constant of the velocity storage, particularly in interaction with gravity signals (3), reflected by a prolonged velocity storage (4) and an inability to suppress post-rotatory nystagmus by head-tilt (5–7). Other clinical findings in isolated nodular lesions include periodic alternating nystagmus (PAN) (6, 7), positional downbeat nystagmus in association with a disturbance in the integration of otolith signals and perverted head-shaking nystagmus (HSN) (7). Also the presence of direction-changing positional apogeotropic horizontal nystagmus (8–10), and a contraversive ocular tilt reaction (2, 7) have been linked to nodular strokes. Besides vestibulo-ocular motor deficits also vestibulo-perceptual impairments may be observed. Specifically, an abolished earth verticality perception has been demonstrated in a single patient with acute heminodular stroke (10).

Isolated nodular infarction, however, is very rare and acute lesions involving the nodulus with or without associated cerebellar structures supported by the medial posterior inferior cerebellar artery (mPICA) (11) may cause an acute vestibular syndrome (AVS), mimicking acute peripheral vestibulopathy (so-called pseudo-vestibular neuritis) (12, 13). Misdiagnosis of AVS may lead to a disastrous result, and many efforts have been made to increase the bedside diagnostic accuracy in distinguishing peripheral from central AVS cases, including the application of the “HINTS plus: Head-Impulse, Nystagmus, Test-of-Skew and new-onset hearing loss” (14, 15) and other exams (16). Of note, head impulse testing (HIT) has been a key examination to differentiate a dangerous stroke from a benign, usually self-limited condition such as an acute peripheral vestibulopathy. In isolated nodular infarction, normal HIT is predicted based on former studies and neuro-anatomical knowledge (7, 13, 17). Likewise, in PICA territory strokes the bedside HIT is usually preserved (2, 17). Mild angular vestibulo-ocular reflex (aVOR)-gain reductions with small corrective saccades (which may go undetected by the naked eye alone) have been reported in a single study on PICA stroke patients using magnetic search coils for quantifying the head-impulse test (18). While in some of these patients the nodulus was affected as well, no isolated nodular stroke cases were included. Noteworthy, reduced horizontal head-impulse gains on the contralesional side have been reported in two patients with ischemic strokes involving the right flocculus (which belongs also to the vestibulo-cerebellum) (19, 20).

With regards to isolated nodular infarctions, two cases series have reported on head impulse testing (7, 13). Specifically, bedside HIT was rated as normal in all patients included, however, these studies did not assess the aVOR quantitatively by using either video head-impulse testing (vHIT) or search-coil techniques and also did not test the vertical canals. Thus, in order to address this area of uncertainty (17), we analyzed the aVOR and associated ocular motor findings in a series of patients with isolated nodular infarction using a vHIT-device. We predicted preserved aVOR of both horizontal and vertical semicircular canals when quantitatively assessed in patients with isolated (hemi)nodular stroke.

## 2. Material and methods

### 2.1. Ethical approval

This study was carried out in accordance with the recommendations of the Institutional Review Board of the Chonnam National University Hospital (Gwangju, South Korea) with written informed consent from all subjects in accordance with the Declaration of Helsinki. The protocol was approved by the Institutional Review Board of the Chonnam National University Hospital (Gwangju, South Korea). The research project was conducted in accordance with university policies, the Personal Information Protection Act of Korea, the Declaration of Helsinki (except for registration in a database), the principles of Good Clinical Practice, the Human Research Act (HRA) and the Human Research Ordinance (HRO). Data will be made available on request from the authors.

### 2.2. Subjects

**Figure 1:**
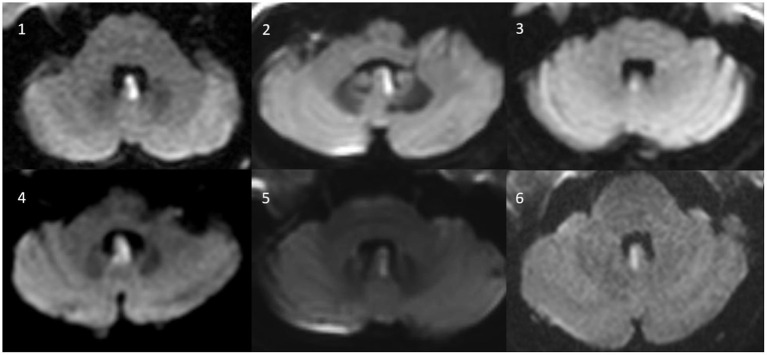
Axial MR-images (diffusion weighted images) of all six patients with isolated (hemi)nodular infarction.

We searched the prospective stroke registry of the Chonnam National University Hospital, Gwangju, South Korea, for patients presenting to the emergency department or the outpatient clinic that received a diagnosis of isolated nodular infarction on magnetic resonance imaging (MRI) including diffusion-weighted imaging (DWI) (see Figure 1). Between January 2015 and December 2021, we retrospectively identified 8 consecutive patients who received quantitative vestibular testing. Two patients were excluded. Reasons for exclusion were pre-existing ophthalmoplegia (right abducens nerve palsy) affecting the results of vHIT (*n* = 1) and previous strokes in the inferior-medial cerebellum and the pons (potentially affecting the ocular motor findings) (*n* = 1). Eventually, six patients with isolated nodular infarction with compatible radiologic findings were enrolled for this study. None of these patients have been previously published.

### 2.3. Experimental setup and paradigm

A structured bedside neuro-otologic examination was obtained in all patients. Video-oculography (VOG; SLMed, Seoul, South Korea, recording frequency = 60 Hz) was performed in a sitting position with fixation removed for the detection of spontaneous (horizontal, vertical, or torsional) nystagmus, positional nystagmus, head-shaking nystagmus and periodic alternating nystagmus for at least 2 min in each patient. For testing of pursuit eye movements, saccades and eccentric gaze holding [looking for gaze-evoked nystagmus (GEN)] visual targets were used. All subjects received a detailed neurologic examination and vHIT after a median of 5 days (range: 1–20 days) after symptom onset.

Imbalance was graded from 0 to III as follows (7): Grade 0 (normal), able to stand on tandem Romberg with the eyes open for 3 s; grade I (mild), unable to stand on tandem Romberg with the eyes open at least for 3 s; grade II (moderate), unable to stand on Romberg with the eyes open at least for 3 s; grade III (severe), unable to stand or sit without support.

Details on vHIT have been described in detail before (21). In brief, vHIT of both the horizontal and vertical canals was obtained using a lightweight, portable VOG device (ICS Impulse; Otometrics, Taastrup, Denmark) (22). We aimed for head velocities between 150 and 200°/s and head displacements of 10–20°. For each canal, 20 valid head-impulses were required.

### 2.4. Data analysis

Both clinical information (presenting symptoms, symptom duration, findings from bedside examination) and results from quantitative testing were retrieved. Gains of the vHIT recordings were analyzed using OtosuiteV 4.0 (Otometrics). This software visualizes all compensatory saccades to ensure accurate characterization. Overt saccades were defined as saccades that occurred in the opposite direction of the head rotation and that reached peak acceleration after the head had stopped moving. Covert saccades, on the other hand, reached peak acceleration before the head had stopped moving (23). Noteworthy, a distinction from early (covert) catch-up saccades (CS) is usually readily possible. Therefore, visual inspection as done for all traces as part of the overall rating of vHIT will ensure that inappropriate traces may still be removed. All vHIT traces were independently reviewed by two experienced neuro-otologists (SHL and AAT). Reviewers were blinded to the clinical findings and the results from MR imaging.

For this study, we relied on the standard aVOR gain calculations from the Otometrics vHIT goggles (OtosuiteV 4.0). This algorithm calculates gain as the ratio of the area under the desaccaded eye-velocity curve (AUC) to the area under the head-velocity curve, corresponding to a desaccaded position gain (22). Thus, the gain of the aVOR was calculated as the ratio of the cumulative slow-phase eye velocity over the cumulative head velocity from the onset of the head impulse to the moment when head velocity crossed zero again (22). We used the cutoff values in aVOR gains as proposed by the manufacturer of the video-goggles (Otometrics), i.e., 0.8 for the horizontal canals and 0.7 for the vertical canals. These values were also in agreement with normative values for a wide range of ages reported (24). The video-head impulse traces were evaluated by the reviewers for reduced aVOR-gain, increased corrective saccades (overt or covert), or a combination of both (25) and an overall rating was provided (“normal” or “abnormal”). Disagreements between the two raters were resolved by discussion and–if needed–by a judgment call of a third rater.

## 3. Results

### 3.1. Clinical and neuro-otologic/neuro-ophthalmologic findings

A total of six patients (4 women, mean age=59.3 ± 9) with isolated nodular infarction on DWI were consecutively enrolled for this study. Five out of six patients (5/6, 83.3%) met the diagnostic criteria for acute vestibular syndrome (AVS) as proposed by the classification committee of the Bárány Society (acute onset of vertigo or dizziness with nausea or vomiting, head-motion intolerance, and unsteadiness) (26), and the remaining sixth patient had an triggered episodic vestibular syndrome (tEVS) (see Table 1 for details). All six patients had normal bedside HIT and did not show GEN. One patient (case 2) showed a skew deviation (with the ipsilesional left eye demonstrating hypertropia). Applying the HINTS battery to those five patients presenting with an AVS, they were found to be central in all subjects. All five patients with AVS had truncal ataxia; three patients had grade II imbalance and two were rated as grade III.

**Table 1:** Clinical and neuro-otologic and neuro-ophthalmologic findings.

**Case**	**1**	**2**	**3**	**4**	**5**	**6**	**Summary**
Age/Sex	49/M	66/F	73/F	57/M	56/F	55/F	
Lesion side	L	L	R	B	L	R	
Clinical feature	AVS	AVS	AVS	AVS	AVS	EVS	AVS (5/6) EVS (1/6)
Delay from symptom onset to bedside testing (hours)	1	1	5	10	1	23	
Delay from symptom onset to quantitative testing (days)	1	20	3	7	1	7	
Bedside HIT	Normal	Normal	Normal	Normal	Normal	Normal	6/6
GEN (bedside only)	-	-	-	-	-	-	No GEN (0/6)
Skew deviation (bedside only)	-	+	-	-	-	-	1/6
Truncal ataxia (bedside only)	++	+++	++	+++	++	-	5/6
SN (bedside and VOG)	-	LBN^*^	Subtle RBN^*^	-	-	-	2/6
PAN (VOG)	-	-	-	-	-	-	No PAN (0/6)
HSN (VOG)	-	LBN	-	RBN/DBN	LBN	-	3/6
PN (VOG)	DCPN (apo)	LBN/DBN	RBN	DCPN (apo)	LBN	DCPN (apo)	6/6
Saccadic eye movements (VOG)	Normal	Normal	NA	Normal	Normal	Normal	Normal (5/5)
Pursuit eye movements (VOG)	Normal gain, SP	Decreased gain, SP	NA	Normal gain, SP	Decreased gain, SP	Decreased gain, SP	Abnormal (5/5)

Truncal ataxia was graded from 0 to +++ according to Moon et al. (7): 0 (normal), able to stand on tandem Romberg with the eyes open for 3 seconds; + (mild), unable to stand on tandem Romberg with the eyes open at least for 3 seconds; ++ (moderate), unable to stand on Romberg with the eyes open at least for 3 seconds; +++ (severe), unable to stand or sit without support.

* Without fixation.

Apo, apogeotropic nystagmus; AVS, acute vestibular syndrome; DBN, downbeat nystagmus; DCPN, direction-changing positional nystagmus; EVS, episodic vestibular syndrome; F, female; GEN, gaze-evoked nystagmus; HSN, head shaking-induced nystagmus; M, male; LBN, left beating nystagmus; NA, not available; PN, positional nystagmus; RBN, right beating nystagmus; SN, spontaneous nystagmus; SP, saccadic pursuit; VOG, video-oculography.

**Figure 2:**
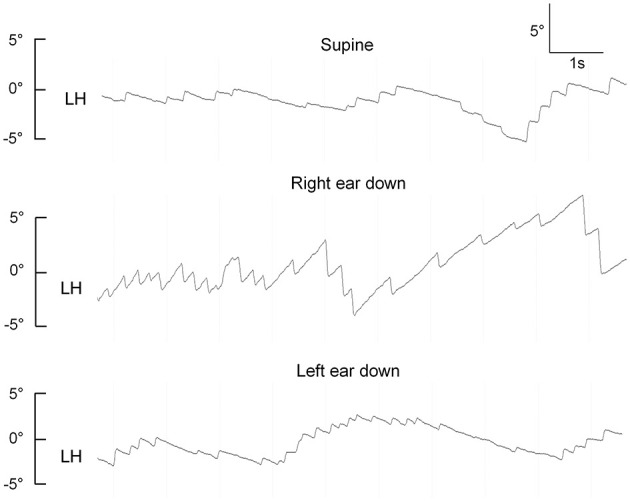
Apogeotropic positional nystagmus in a patient with an isolated left-sided heminodular infarction (case 1), demonstrating horizontal eye position from the left eye (LH). Upward excursions reflect eye movements toward the right side, downward excursions are consistent with eye movements to the left.

Two out of six patients presented with horizontal spontaneous nystagmus (SN) that was beating to the ipsilesional side. Horizontal head-shaking triggered a nystagmus in two patients (horizontally beating to the ipsilesional side in one patient and right-beating/down-beating in the other patient), and augmented a horizontal SN (with beating direction being unchanged) in a third patient. Positional testing was performed in a sequential manner (pitch-plane testing, straight head-hanging (SHH), Dix-Hallpike maneuvers, and supine roll tests). In one patient, persistent positional downbeat nystagmus (pDBN) with a horizontal component as well was evoked by SHH. In three patients (3/6, 50%), direction-changing positional nystagmus (DCPN) with an apogeotropic pattern was observed during supine roll testing (see Figure 2 for an illustrative case). Ocular motor testing for saccades and pursuit eye movements was performed in five patients. Saccades were rated as normal in all tested subjects, but pursuit was abnormal because of either decreased pursuit gain or interleaved saccadic eye movements.

### 3.2. Quantitative assessment of the angular VOR by use of vHIT

**Figure 3:**
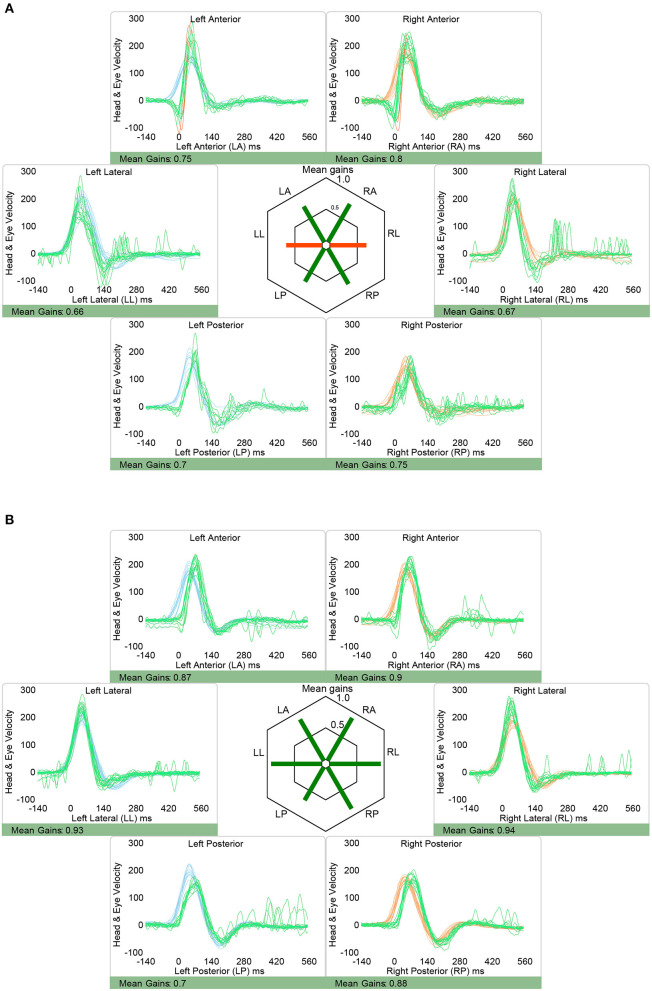
Representative video head impulse testing from two patients with isolated nodular infarction, demonstrating slightly reduced aVOR-response in the horizontal canals in one patient [**(A)**, case 2] and preserved aVOR-response in all six semicircular canals in the other illustrative patient [**(B)**, case 6]. Note that in both patients vHIT responses were judged as overall normal, however, it is acknowledged that the saccades observed when testing the right horizontal canal in case 2 may be considered clinically relevant also, reflecting minor aVOR impairment for this canal. Eye velocity traces (in green) and head velocity traces (in red for testing the right vestibular organ and in blue for assessing the left vestibular organ) are plotted against time. Note that eye velocity traces were inverted for better visualization and comparison with the head velocity traces and that gain was calculated as the ratio of the area under the desaccaded eye-velocity curve to the area under the head-velocity curve, corresponding to a desaccaded position gain. Summary plots in the center illustrate average individual vestibulo-ocular reflex (VOR)-gains ± 1SD for all six canals.

vHIT showed normal aVOR-gains and no catch-up saccades except for one patient (case 2). In case 2, there were mildly decreased gains in both horizontal canals (right 0.66, left 0.67; Table 2). However, catch-up saccades were considered as not clinically relevant and the overall patterns of the aVOR-response was rated as normal by both reviewers. Illustrative cases (case 2 and case 6) are shown in Figure 3.

**Table 2:** vHIT gains in all six patients.

**Case**	**Age/gender**	**Lesion side**	**Ipsilesional vHIT gains**			**Contralesional vHIT gains**		
			**HSC**	**ASC**	**PSC**	**HSC**	**ASC**	**PSC**
1	49/M	L	1.05	0.92	0.89	1.29	0.92	0.89
2	66/F	L	0.66	0.75	0.70	0.67	0.80	0.75
3	73/F	R	0.96	0.92	0.85	0.88	0.82	0.77
4	57/M	B (L > R)	1.02	0.72	0.91	1.19	0.73	0.90
7	56/F	L	0.97	0.81	0.71	1.12	0.81	0.77
8	55/F	R	0.94	0.90	0.88	0.93	0.87	0.70

ASC, anterior semicircular canal; B, bilateral; HSC, horizontal semicircular canal; L, left, PSC, posterior semicircular canal; R, right; vHIT, video-head-impulse test.

## 4. Discussion

Isolated (hemi)nodular strokes as underlying cause of acute dizziness are rare, thus there are still gaps of knowledge in the clinical presentation of affected patients. This includes the integrity of the horizontal and vertical angular vestibulo-ocular reflex (aVOR), with previous publications being restricted to bedside testing of the horizontal aVOR. Thus, the primary aim of this case series was to characterize the spectrum of clinical presentations in isolated (hemi)nodular strokes. In summary, we demonstrated deficits in both postural control, volitional eye movements (pursuit, saccades), ocular stability holding in primary position and during positional testing and after head-shaking. Detailed and quantitative, video-based testing of the horizontal and vertical aVOR confirmed preliminary findings at the bedside, emphasizing a preserved integrity of the aVOR in (hemi)nodular strokes. Likewise, eccentric gaze holding remained intact in these patients. In the following, we will discuss the main findings in more detail and review the literature.

### 4.1. Clinical manifestation of isolated nodular infarction

The PICA supplies the lateral medulla, the inferior cerebellar peduncle, and the cerebellar nodulus and uvula (11). Infarction in the distribution of the distal PICA may cause acute vertigo and nystagmus that mimics an acute peripheral vestibular lesion. These symptoms and signs are probably due to a central vestibular imbalance created by asymmetric infarction in the vestibulo-cerebellum, which normally has a tonic inhibitory effect on the vestibular nuclei (2).

As suspected, acute infarction restricted to the cerebellar nodulus mostly resulted in a clinical presentation meeting criteria for an acute vestibular syndrome (AVS) (26). Since other obvious focal neurologic signs were not observed (excluding truncal ataxia with imbalance and subtle ocular motor abnormalities), all our six patients presented with an isolated vestibular syndrome (acute or episodic), making the differential diagnosis from vestibular neuritis, an AVS with benign course due to peripheral origin, even more challenging. Applying the HINTS battery, however, a three-step bedside diagnostic algorithm to differentiate dangerous central AVS (pseudo-VN) from benign peripheral AVS [e.g., vestibular neuritis (VN)] (14), it pointed to a central cause of AVS (normal bedside HIT in 5/5 and skew deviation in 1/5) in all five patients with AVS in our case series. Also, there was an imbalance of at least moderate severity, which suggested more frequently a central pathology than a peripheral one (27, 28).

Of interest, one patient presented with a triggered episodic vestibular syndrome (tEVS), with vertigo being evoked by positional changes, resembling rather benign paroxysmal positional vertigo (BPPV) than vestibular neuritis. This patient (case 6) showed apogeotropic positional nystagmus mimicking horizontal canal BPPV with cupulolithiasis or short-arm canalolithiasis (29, 30). However, the patient did not improve after repetitive canalith repositioning maneuvers and she had abnormal pursuit eye movements. Both findings suggested a central pathology rather than BPPV having a benign course. Noteworthy, apogeotropic horizontal positional nystagmus in supine-roll testing is much more likely to be of central origin than classic upbeat-torsional nystagmus seen in Dix-Hallpike maneuver (31).

### 4.2. Normal video-HIT in isolated (hemi)nodular infarction

Using quantitative, video-based head-impulse testing of both the horizontal and vertical semicircular canals, we confirmed and extended findings previously reported at the bedside (7), emphasizing a preserved aVOR in isolated (hemi)nodular stroke. This was reflected by both normal vHIT gains and absence of clinically relevant compensatory overt / covert catch-up saccades.

Noteworthy, the nodulus/uvula may also enhance aVOR-gains during HIT (17). All nodulus-target neurons are tuned to vestibular stimuli, and most are insensitive to eye movements. Such non-eye-movement neurons are thought to project to vestibulo-spinal and/or thalamo-cortical pathways. Less than 20% of the nodulus/uvular target neurons respond to both vestibular and eye movement signals, suggesting that the nodulus/uvula can also directly influence vestibulo-ocular pathways (32).

The nodulus and the ventral uvula govern the velocity-storage mechanism of the aVOR to optimize its properties for low-frequency (sustained) rotations (4, 8, 33). In contrast, the HIT reflects a high-frequency stimulus, assessing the aVOR-response in a different frequency range. Therefore, according to our results, (hemi)nodular lesions seem to have little effect on the aVOR as assessed by the bedside HIT / vHIT.

### 4.3. Positional nystagmus, especially direction-changing apogeotropic pattern

The nodulus and the ventral uvula are also important for the generation of normal vestibulo-ocular responses to linear movements (translations), probably acting as the integrator of otolithic inputs (34). Clinical lesions involving the nodulus and uvula may also cause downbeat nystagmus (especially during positional testing), horizontal central positional nystagmus, and produce variants of skew deviation and abnormal ocular counterroll, which implies a central otolithic imbalance. We observed an apogeotropic central positional nystagmus (CPN) in 3/6 patients, being consistent with previous reports on positional nystagmus in patients with isolated nodular stroke (29). Apogeotropic CPN can result from the summation of incorrectly interpreted canal-induced nystagmus and gravity-induced nystagmus in both ear-down positions. A lesion disrupts the pathway providing the estimated direction of gravity to the rotational feedback loop and also produces a positive bias toward the nose along with a naso-occipital axis, the compensatory rotational feedback would generate a constant horizontal, apogeotropic gravity-induced nystagmus (29). Noteworthy, with an apogeotropic CPN being observed in 2/5 patients with heminodular stroke in our case series, this confirms previous observations, that partial loss of the nodulus is sufficient to cause apogeotropic CPN (7, 10).

### 4.4. Other vestibulo-oculomotor symptoms and signs

Various other ocular motor and vestibular signs were observed in our case series, including ispilesional beating HSN (3/6 patients), misdirected HSN (cross-coupled HSN) with a DBN component (1/6 patients), positional DBN during SHH (1/6 patients), and contraversive skew deviation (1/6 patients). Impaired pursuit eye movements (decreased gains and/or saccadic pursuit) were observed in all patients.

But several vestibulo-oculomotor signs such as PAN were not observed as expected in our case series. Ischemic stroke lesions may be different from targeted, iatrogenic experimental lesions in monkeys. Furthermore, in our case series 5/6 patients had heminodular strokes only and a single patient demonstrated a bilateral ischemic stroke of the cerebellar nodulus. Previously, PAN has been reported only in patients with bilateral isolated nodular infarction (6, 7), whereas it was not seen in patients with heminodular stroke (7, 10).

### 4.5. Limitations section

In one patient (#2) gathered saccades could be depicted about 100 ms after the head-impulse was applied for testing of the right horizontal canal. While both the built-in algorithm (OtoSuite V4, Otometrics) and the two reviewers judged these saccades as not clinically relevant, their clinical significance remains debated especially in combination with a slightly reduced aVOR gain. Thus, a mildly impaired aVOR-response for this canal cannot be fully excluded, however, would be on the side opposite to the heminodular stroke in this patient. Therefore, we consider it unlikely, that these mild changes reflect a true aVOR impairment.

## 5. Conclusions

Providing both standardized bedside and quantitative ocular motor and vestibular testing in patients with isolated (hemi)nodular stroke, we confirmed and extended previously reported deficits in this rare condition. Specifically, at the bedside impairments in both postural control, pursuit eye movements, ocular stability (in primary position and during positional testing) and after head-shaking were identified. Detailed and quantitative, video-based testing of the horizontal and vertical aVOR confirmed preliminary findings at the bedside, emphasizing preserved integrity of the aVOR in (hemi)nodular strokes. Noteworthy, heminodular lesions were found to be sufficient to trigger positional nystagmus. When presenting as triggered EVS (as in 1 out of 6 patients in our cohort), distinction from benign paroxysmal positional vertigo may be challenging.

## Data availability statement

The raw data supporting the conclusions of this article will be made available by the authors, without undue reservation.

## Ethics statement

The studies involving human participants were reviewed and approved by Institutional Review Board of the Chonnam National University Hospital (Gwangju, South Korea). The patients/participants provided their written informed consent to participate in this study.

## Author contributions

S-HL: conception of the work, data collection, data analysis, interpretation of data for the work, drafting the manuscript, and revising the work critically for important intellectual content. J-MK: interpretation of data for the work, drafting the work, and revising it critically for important intellectual content. J-TK: data collection, interpretation of data for the work, and revising the work critically for important intellectual content. AT: conception of the work, data analysis, interpretation of data for the work, drafting the work, and revising it critically for important intellectual content. All authors approved the final version of the manuscript and agreed to be accountable for all aspects of the work in ensuring that questions related to the accuracy or integrity of any part of the work are appropriately investigated, resolved, and confirm that all persons designated as authors qualify for authorship, and all those who qualify for authorship are listed.
